# pH-Responsive Alginate/Chitosan Gel Films: An Alternative for Removing Cadmium and Lead from Water

**DOI:** 10.3390/gels10100669

**Published:** 2024-10-19

**Authors:** Silvia Carolina Moreno-Rivas, María José Ibarra-Gutiérrez, Daniel Fernández-Quiroz, Armando Lucero-Acuña, Alexel J. Burgara-Estrella, Paul Zavala-Rivera

**Affiliations:** 1 Department of Chemical Engineering and Metallurgy, University of Sonora, Hermosillo 83000, Mexico; carolina.moreno@unison.mx (S.C.M.-R.); majoibarragtz@gmail.com (M.J.I.-G.); armando.lucero@unison.mx (A.L.-A.); 2 Department of Physics Research, University of Sonora, Hermosillo 83000, Mexico; alexel.burgara@unison.mx

**Keywords:** biopolymers, potentially toxic metals, gel films

## Abstract

Biosorption, a non-expensive and easy method for removing potentially toxic metal ions from water, has been the subject of extensive research. In this context, this study introduces a novel approach using sodium alginate and chitosan, versatile biopolymers that have shown excellent results as biosorbents. The challenge of maintaining high efficiencies and reuse is addressed by developing alginate/chitosan-based films. These films, prepared using solvent casting and crosslinking methods, form a hydrogel network. The alginate/chitosan-based films, obtained using the eco-friendly polyelectrolyte complex method, were characterized by FTIR, SEM, TGA, and DSC. The study of their swelling pH response, adsorption, and desorption behavior revealed promising results. The adsorption of Pb was significantly enhanced by the presence of both biopolymers (98%) in a shorter time (15 min) at pH = 6.5. The adsorption of both ions followed a pseudo-second-order kinetic and the Langmuir isotherm model. The desorption efficiencies for Cd and Pb were 98.8% and 77.6% after five adsorption/desorption cycles, respectively. In conclusion, the alginate/chitosan-based films present a highly effective and novel approach for removing Cd and Pb from water, with a promising potential for reuse, demonstrating their strong potential in potentially toxic metal removal.

## 1. Introduction

Water pollution is an increasing worldwide concern. Potentially toxic metals (PTMs) are the most relevant drinking water pollutants. In particular, cadmium (Cd) and lead (Pb) imply environmental and health risks even at low concentrations. Cd exposure is linked to various adverse effects, including pancreas, breast, prostate, bladder, and lung cancer [1,2]. On the other hand, exposure to Pb is associated with central nervous system damage, liver and lung diseases, anemia, skeletal growth, and cancer [3,4]. These metals come from wastewater from different industries such as mining, paper industry, agriculture, and metal planting [5]. Therefore, removing these pollutants from water is one of the leading environmental remediation goals.

Different techniques, such as coagulation, photocatalysis, membrane filtration, precipitation, and adsorption, have been studied to remove these metal ions from water [6,7,8]. The biosorption method has gained significant attention in the last decade because it is simple, clean, low-cost, and efficient. It has been applied to recover metals in diluted solutions with low investment costs [9]. It employs renewable sorbents, which can be extracted from cultures and secondary sources such as algae, fungi, bacteria, and agro-industrial and aquaculture wastes [10,11,12].

Biosorption is a passive adsorption uptake process in which the pollutant molecules attach to the biomass surface depending on its physicochemical characteristics, such as chemical composition, surface charge, hydrophobicity, etc. [13]. In the case of PTMs, the biosorption process is positively affected by the presence of functional groups in the biosorbent [14]. For example, dead yeast biomass successfully removed uranium ions from an aqueous solution due to the presence of carboxyl, amino, and phosphoryl groups [15]. In another study, Hu et al. [16] confirmed that the strontium and cession ions bind to the sulfate, carboxyl, and amino groups in the marine algae (*Undaria pinnatifida*) biomass. In addition, the pH also plays an essential role in PTMs biosorption since it may affect the biosorbent structure and, therefore, metal uptake [9].

Hydrogels in different forms have been investigated as potential biosorbents. They are crosslinked three-dimensional networks with a hydrophilic nature, able to adsorb large amounts of water [16]. Hydrogel films have mainly been studied due to their homogeneous surfaces, high flexibility, and porosity [17]. The applicability of hydrogels depends on their source, composition, environmental stimuli, crosslinking, property, configuration, and ionic charge [18].

In this sense, biopolymers are an excellent option for obtaining hydrogel films and removing ions due to positive and negative charge functional groups (amide, carbonyl, carboxyl, hydroxyl, etc.) [12]. In addition, they are non-toxic and biodegradable because they are obtained from natural sources [16]. Among the biopolymers used for the adsorption of PTMs are lignin, cellulose, xanthan, chitosan, and alginate, among others [19].

Alginate is a biopolymer extracted from brown algae, conformed by mannuronic (M) and guluronic (G) acid units arranged in different MG blocks throughout its structure [20]. This biopolymer forms a gel in the presence of multivalent cations, such as Ca^2+^, due to the “egg-box model” mechanism produced by ionic interactions between guluronic acid residues from two or more alginate chains and the cations [21]. It has been used as an anionic adsorbent with remarkable adsorption efficiency. It has also been mixed with other materials to enhance its removal performance because alginate dissolves in water and alkaline solutions [22]. For example, alginate beads have been used to remove Pb from aqueous solution at a pH of 5.0, presenting a maximum adsorption capacity of 261.9 mg g^−1^ during a contact time of 90 min [23]. On the other hand, chitosan is an adsorptive cationic polymer primarily obtained from the deacetylation of chitin molecules, the major structural component of crustacean shells and the cell walls of fungi and yeast [24,25]. It has shown an affinity towards pollutants in wastewater because of its amino and hydroxyl groups [26]. Regardless, chitosan suffers from poor stability and low mechanical strength. To overcome these limitations, structural and chemical modifications, such as cross-linking, which strengthens chitosan by bridging polymer chains and functional groups, have been investigated. However, it may reduce the adsorption uptake [27].

In this sense, forming a polyelectrolyte complex (PEC) between alginate and chitosan represents a promising alternative due to its ease of use at the laboratory scale, biodegradability, biocompatibility, and improved sorption ability at several pH intervals [20]. It also allows tailoring to different forms and sizes, including hydrogel films [28]. This PEC has been studied mainly in bead shapes, with and without crosslinkers, and has been previously investigated for removing PTMs such as Cu, Hg, Zn, Cd, and Pb [29]. Nevertheless, maintaining high efficiencies and short contact times and reusing these biosorbents is still challenging [30,31].

The present study aimed to synthesize alginate/chitosan-based gel films through an easy, effective, and eco-friendly method for removing Cd and Pb from water. It obtained its physicochemical characteristics by FTIR, SEM, TGA, DSC, and pH-dependence swelling behavior analysis.

## 2. Results and Discussion

In this work, we prepared calcium alginate (Alg-Ca), chitosan crosslinked with TPP (Cs-TPP), and alginate/chitosan (Alg/Cs) gel films using the ionic crosslinking method. A crosslinked bath containing chitosan and calcium chloride was used to obtain alginate/chitosan-based films (Alg/Cs). The cross-linking process occurred spontaneously by interacting -COO^-^ ions from the alginate with the chitosan [-NH_3_^+^] groups and multivalent Ca^2+^ ions. It is known that the formation mechanism of the Alg-Ca crosslinked system is driven by the ionic interaction between alginate chains and calcium ions, forming an “egg-box” model. In the presence of chitosan, the ionic gelation process is expected to occur concomitantly with the complexation of the polyelectrolyte with chitosan [-NH_3_^+^] groups [32]. In addition to the ionic interactions of the carboxyl group of alginate and amino groups, intramolecular and intermolecular hydrogen bonding of both biopolymer structures may occur [33,34]. In the present study, introducing chitosan as a second constituent in the gel film opens new possibilities for the remotion of metal ions with different charges, as discussed in detail below.

### 2.1. Fourier Transform Infrared Spectroscopy

**Figure 1:**
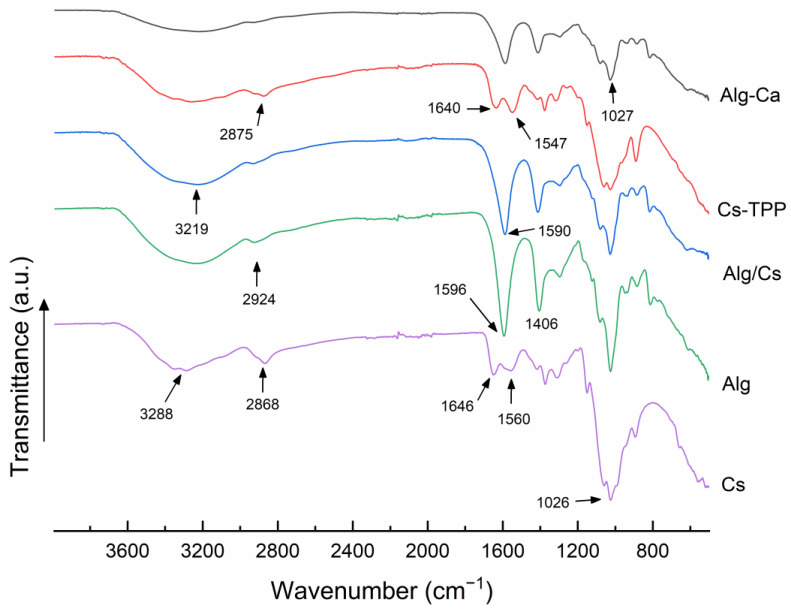
FTIR spectra of a calcium alginate film, chitosan crosslinked with TPP film, calcium alginate/chitosan film, sodium alginate, and chitosan.

FTIR analysis allows the identification of the main functional groups in each biopolymer and changes in absorption patterns to confirm interactions. Figure 1 shows the spectra of Alg-Ca, Cs-TPP, Alg/Cs, chitosan (Cs), and sodium alginate (Alg). Spectra of Alg-Ca films and sodium alginate showed a broad absorption band at 3220 cm^−1^, associated with stretching of -OH groups [35]. The absorption band at 1596 cm^−1^ corresponds to the asymmetric stretching of the -COO group, whereas around 1406 cm^−1^, the symmetric vibration of the same group is identified [23,36]. At 1026 cm^−1^, the asymmetric stretching of C-O-C bonds is displayed. In contrast, between 880 and 1000 cm^−1^ is the region where the C-O bond vibrates, associated with mannuronic acid units of alginate structure [37].

The spectra of Cs-TPP films and Cs also showed a characteristic broad band of -OH stretching (3288 cm^−1^). Amide I and Amide II vibration bands were located at 1646 cm^−1^) and 1560 cm^−1^, respectively. The same peaks show a slight shift in Cs-TPP, corresponding to electrostatic interactions between amino and phosphate groups of TPP [38]. On the other hand, the predominant bands in the spectrum of Alg/Cs-based film correspond to the typical bands of Alg-Ca film. The vibration band at 1590 cm^−1^, concerning the stretching of the -COO group, displays a perceivable change in intensity, which is possibly due to an increase in the electrostatic attraction exerted on this functional group by the introduction of cationic chitosan macromolecules [39]. Hamedi et al. [24] also observed this change in alginate–chitosan films for drug delivery. Also, a slight increase is observed around 2900 cm^−1^ in Alg/Cs compared to Alg-Ca, concerning the C-H stretching of carbon chains of both biopolymers [40]. These findings demonstrate concomitant electrostatic interaction between alginate and chitosan/calcium. According to the procedure performed to obtain gel films, alginate may be mostly cross-linked with Ca^2+^ ions, with a low chitosan concentration in the film, whose chains may probably be predominantly trapped in the Alg-Ca network.

### 2.2. Surface Morphology of Gel Films

**Figure 2:**
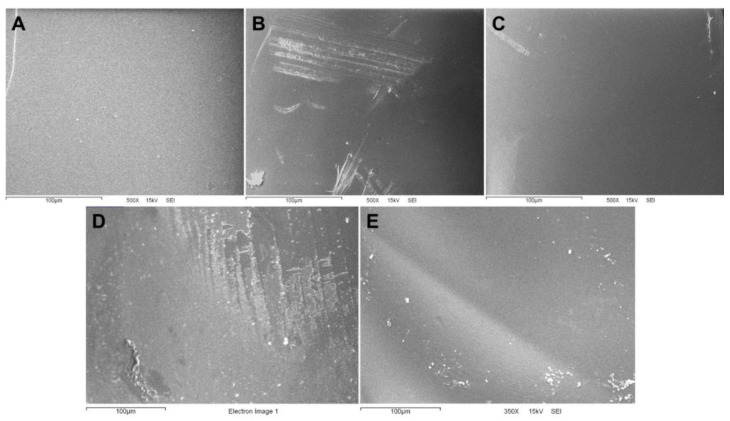
Scanning electron microscopy (SEM) surface images of the gel films: (**A**) calcium alginate, (**B**) chitosan-TPP, and the calcium alginate–chitosan films (**C**) before adsorption, (**D**) after Cd adsorption, and (**E**) after Pb adsorption, at *t* = 15 min.

Figure 2 displays the surface images of Alg-Ca (A), Cs-TPP (B), and Alg/Cs dry gel films before adsorption (C) and after adsorption of Cd (D) and Pb (E). Cs-TPP film (Figure 2B) shows surface scratches, consistent with a more fragile and brittle structure attributed to chitosan films [41,42,43]. For Ca-Alg and Alg/Cs films, surfaces have a more homogeneous and smooth appearance; these characteristics have been previously attributed to alginate-based films by other authors [44,45]. On the other hand, the films exhibited apparent microstructural changes after the Cd (Figure 2D) and Pb biosorption (Figure 2E)—surfaces seem less homogeneous and exhibit particle depositions. The literature has described analogous changes for other biosorbents and their relationship with metal interactions with biopolymer chains. In contrast to similar biosorbents, film continuity is maintained after adsorption, which may compromise the integrity of the films [46,47]. An EDS elemental analysis of biopolymeric films after the biosorption process confirmed the inclusion of Cd and Pb into the hydrogel network. These results are consistent with changes observed by SEM.

**Figure 3:**
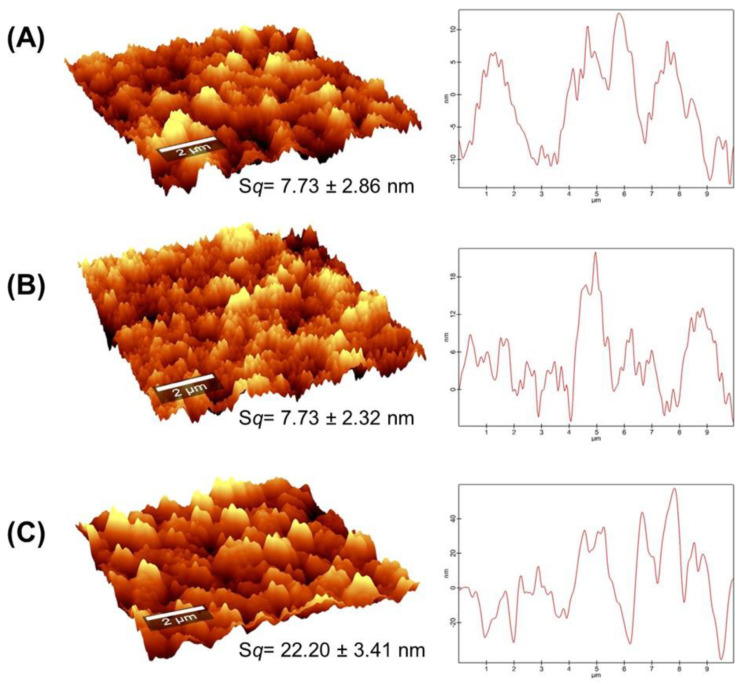
Atomic force microscopy (AFM) of Alg/Cs surface films: (**A**) before adsorption, (**B**) after Cd adsorption, and (**C**) after Pb adsorption, at *t* = 15 min.

Film rugosities were analyzed using atomic force microscopy (AFM) to assess the surface changes observed by SEM. Figure 3 shows the surface images and profiles for Alg/Cs films before and after Cd and Pb adsorption. Root mean square roughness (*Sq*) for Alg/Cs-based films before metal adsorption was 7.73 nm. This roughness value may be attributed to the presence of Cs in the film surface. Simpliciano et al. [48] also reported a smoother surface after Cs coating of crosslinked alginate films by AFM analysis. Other authors have found similar roughness for the Cs film surface [49,50]. Meanwhile, the calcium alginate surface is usually rougher, mainly associated with crosslinker concentration and viscosity [51,52].

The *Sq* value did not change after Cd adsorption. Nevertheless, surface rugosity increased after Pb removal (*Sq* = 22.20 nm). This notable change is presumably related to the adsorption process of each metal since Pb has a higher affinity for ion exchange with Ca^2+^ involved in alginate crosslinking; this probably leads to a structural change in the egg box model due to the higher ionic radius of Pb (1.19 Å) compared to Ca^2+^ (0.99 Å). In this sense, Mousa et al. [53] found a lower Ca^2+^ content after Pb adsorption on chitosan-coated calcium alginate beads, suggesting an exchange between these ions. Also, Milosavljević et al. [49] observed similar surface microstructural changes in chitosan-based hydrogels with itaconic and methacrylic acid after Zn^2+^ adsorption. On the other hand, Cd ionic radius (0.97 Å) is similar to Ca^2+^; therefore, in an ionic exchange of this metal, it is likely that the structure experiences non-quantifiable alterations to its surface roughness, as evidenced by this analysis.

### 2.3. Thermal Properties of Gel Films

**Figure 4:**
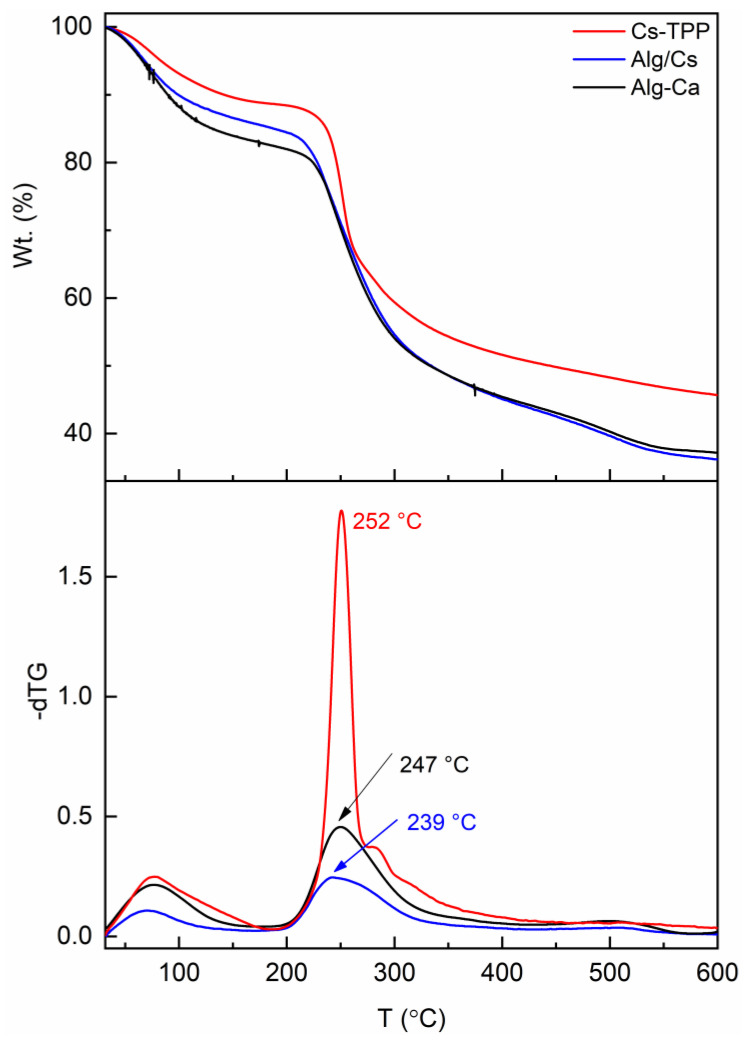
TGA and dTG curves of biopolymeric films.

Figure 4 displays the thermogravimetric (TGA) and differential thermogravimetric (DTG) curves for Cs-TPP, Alg-Ca, and Alg/Cs gel films. The TGA curves of three samples display two thermal steps. The former occurs below 160 °C, which is associated with the dehydration of polysaccharides. Water can be associated in the polymer chain in free or bonded form. Regarding alginate, accessible water is liberated below 60 °C, water linked by dipole-dipole interactions is released until 120 °C, and the water bonded to the -COO- due to ion-dipole interactions is discharged until 160 °C [54]. Slight differences in the amount of water released in each film can be observed, which was most notable for the alginate film (17%). It is possible to observe a slight reduction in the amount of water contained in the Alg/Cs film (14%) compared to that of alginate, which is accompanied by a decrease in the temperature range (until 110 °C) at which this thermal event occurs. This behavior may be linked to a significant reduction in available -COO- ions in the alginate chain due to interactions with the chitosan molecule.

The second thermal event occurs between 200 and 350 °C and exhibits a remarkable mass loss. This step is attributed to polymer degradation involving depolymerization [55]. The maximum degradation temperature in Alg/Cs films can decrease slightly compared to Cs-TPP films. This can be associated with decreased cooperative H-bonding along the chitosan backbone [56] caused by the PEC formation.

The third thermal step occurs above 350 °C and is attributed to the non-oxidative decomposition of the molecules propagated through the degradation of the polysaccharides [54].

**Figure 5:**
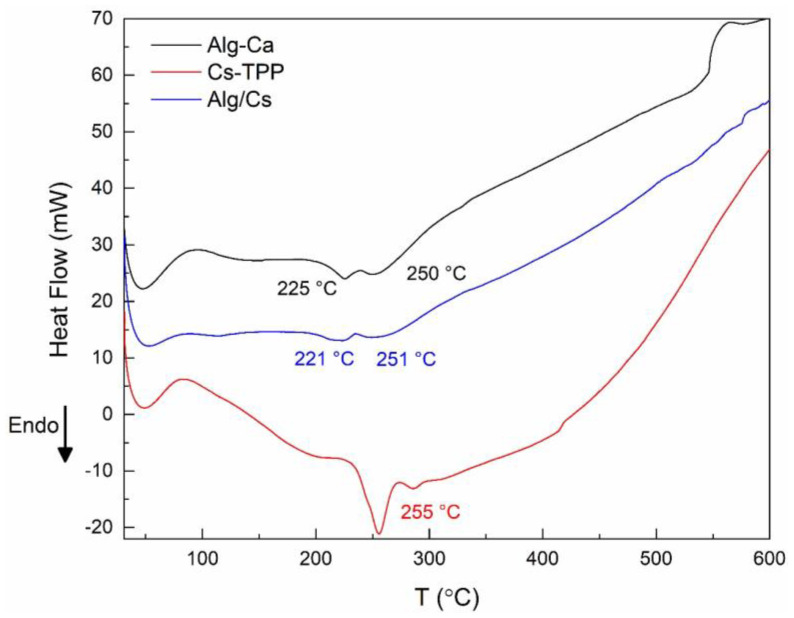
DSC curves of biopolymeric films.

Figure 5 shows the DSC curves for Cs-TPP, Alg-Ca, and Alg/Cs gel films. The DSC showed the presence of three peaks in all samples. The first endothermic peak is below 80 °C, linked with the water release. This event is dependent on the sample. Alg-Ca and Alg/Cs films exhibited endothermic peaks around 225 °C and 221 °C, respectively. This behavior corresponds with the melting of the crystalline structure of calcium alginate. Regarding the Cs-TPP film, its DSC curve displays an endothermic solid peak at 255 °C, attributed to the melting of the crystalline structure of the chitosan, and TPP crosslinked chain [57].

### 2.4. Swelling Behavior of Gel Films

**Figure 6:**
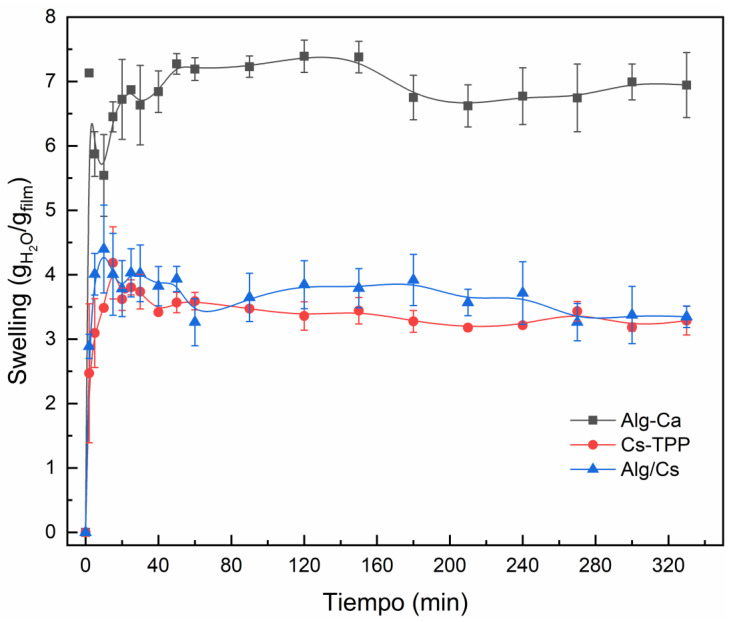
Swelling kinetics of biopolymeric films.

Figure 6 shows the dependence of the swelling degree of the Alg-Ca, Cs-TPP, and Alg/Cs biopolymeric films on time. It is possible to notice that all samples reach swelling equilibrium in less than 10 min, typical of the high porosity characteristic for cross-linked polymeric matrices. Under pre-equilibrium conditions, an abrupt increase in swelling is observed for all samples, and subsequently, the amount of water imbibed in the film decreases until equilibrium is reached. This behavior may be caused by an initial increase in the free water molecules embedded in the capillary pores of the gel [58].

The Alg-Ca film shows the highest water uptake compared to the other films. Jin et al. [59] found that the swelling behavior of alginate hydrogels is related to the degree of viscosity of the sodium alginate. High viscosity is linked to higher swelling. High-viscosity SA possesses a higher proportion of guluronic acid units, increasing the availability of the carboxylic acid group present in the guluronic acid, which readily interacts with the water molecules, improving the water uptake properties [60,61]. In addition, lower calcium concentration for hydrogel crosslinking has been related to an increased swelling index, similar to the concentration applied for these films [62].

Higher water adsorption is proportional to the expansion of the hydrogel and is related to hydrophilic groups within polymeric structures. However, if the hydrogel’s structure is compact, fewer water molecules will permeate [63,64]. In this sense, the Alg/Cs-based films exhibited significantly lower water retention capacity than the Alg-Ca film. This behavior may be possibly associated with an increase in the cross-linking sites of the polymeric matrix. The introduction of the positively charged chitosan chains in the polymeric matrix promotes the formation of a polyelectrolyte complex with the alginate -COO^-^ groups. These electrostatic interactions are in addition to those formed by Ca^2+^ ions. Thus, it is likely that the concomitant cross-linking of alginate with chitosan/calcium chloride favors the formation of a denser reticular meshwork, diminishing the film permeability [65].

**Figure 7:**
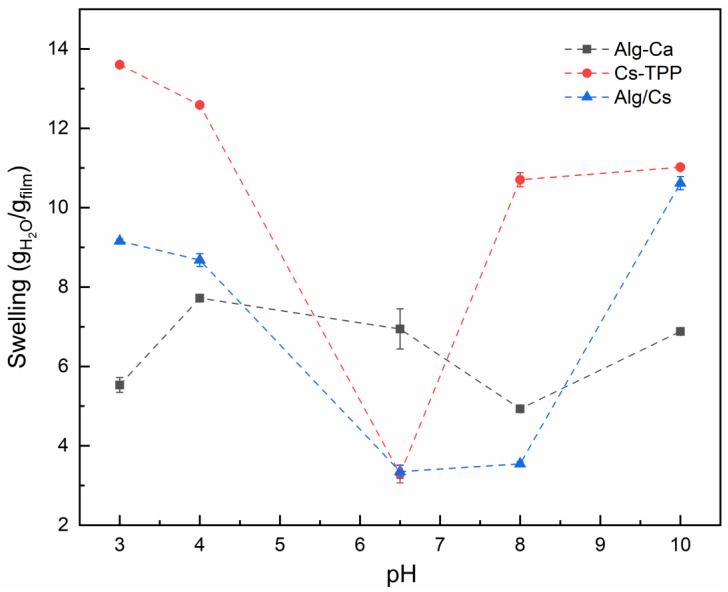
pH-dependence swelling behavior of gel films. Equilibrium time: 5 h.

This behavior is also affected by the pH of the aqueous medium. Figure 7 shows the films’ swelling capacity dependence on the pH. For Alg-Ca and Alg/Cs-based films, low pH causes the calcium ions to dissociate and form calcium phosphate salts, diminishing the interaction with the alginate matrix, which may cause structural changes [66]. Also, the ionization of alginate at low pH generates electrostatic forces between alginate chains, increasing water uptake [67]. For Cs-TPP films, low pH is also associated with a swelling increase, as seen in Figure 6. This is explained by the protonation of amino groups, leading to a relaxation of the hydrogel network and a higher water uptake. At higher pH values (pH 6–7), the amine groups are deprotonated, pushing back the polymer chains by repulsion, which results in a contraction of the polymer matrix, causing a restriction over the water absorption. The Alg/Cs-based film exhibited a lower swelling degree at pH 6.5 than the Alg-Ca film. The degree of crosslinking is associated with a reduced swelling rate because of the compacting of the hydrogel structure, which forms a polyelectrolyte complex. In a similar study, the swelling degree of alginate–chitosan beads was assessed, showing that water uptake increased at low pH at 240 min [68]. At a lower pH, the Alg/Cs-based film decreases its swelling ratio compared to Cs-TPP films, which may indicate a less relaxed network and, therefore, an increase in structural maintenance of gel film. These results demonstrate the swelling capacity of the material at a different pH; this can be interpreted as the capacity of the polymeric matrix to trap substances in a pH range from 3 to 10. The above may be relevant for removing substances from contaminated water since the pH of wastewater can vary depending on the type of industry [9].

### 2.5. Metal Adsorption Analysis

The Cd and Pb removal efficiencies of the biopolymeric films are presented in Figure 7.

**Figure 8:**
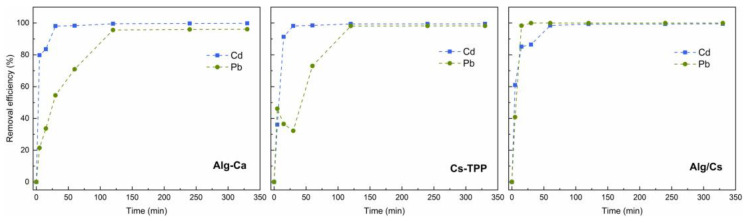
Effect of contact time in cadmium and lead removal efficiency of calcium alginate, chitosan-TPP, and calcium alginate–chitosan films.

The Alg-Ca film shows higher and faster removal efficiency for Cd than Pb ions. As shown in Figure 8, the highest Cd removal was achieved after 30 min (98.1%). On the other hand, Pb has a lower uptake, reaching maximum adsorption after 120 min. Different steps in divalent PTM adsorption onto alginate have been previously elucidated by Mohammed et al. [69]. Thermodynamically, electrostatic interactions occur faster, followed by ion exchange. Also, even when Cd and Pb may interact with carboxylic alginate groups, each ion has different affinities for alginate units. Cd has a higher affinity for mannuronic units, while Pb for guluronic units. This may indicate that the faster Cd adsorption is mainly due to electrostatic interactions with mannuronic units [69]. Pb, which has a higher affinity for guluronic units because of its higher electronegativity, may induce an ionic exchange with Ca^2+^ ions since guluronic blocks are involved in alginate crosslinking. Similar behavior was observed for the Cs-TPP film, with a slower Cd and Pb uptake than the Alg-Ca films. This may be due to the TPP crosslinking reducing the availability of adsorption groups since some repulsion sites may be formed [70].

The Alg/Cs-based film uptake values increased proportionally to the contact time. Mossout et al. [71] observed similar behavior for Cd removal by chitosan-based biosorbent in short times. For the case of Pb, the uptake percentage was significantly higher after 15 min for the Alg/Cs-based film (98.4%) compared to the Alg-Ca (33.7%) and Cs-TPP (36.5%) films separately. The higher uptake of Pb onto Alg/Cs-based film could be attributed to the simultaneous presence of amine, hydroxyl, and carboxylic groups from alginate and chitosan biopolymers [72]. Also, in Alg/Cs films, there is no presence of TPP molecules, which could impact ion uptake. In addition, the smaller hydrated ionic radius and hydration energy of Pb (0.401 nm and −1481 kJ/mol) compared to Cd (0.426 nm, −1807 kJ/mol) could be attributed to the increase in Pb adsorption in a shorter time in Alg/Cs compared to other films. A lower hydrated ionic radius and hydration enthalpy are associated with a higher possibility of interacting with the biosorbent surface, increasing adsorption [73,74]. Also, carboxylic groups may chelate positively charged metal ions, but there is no evidence of chelation formation in FTIR spectra of films after adsorption (Figure S1). In addition, at pH values above 5.0, the deprotonation of amines and hydroxyl groups increases, which can result in the binding with positively charged ions [75]. These deprotonated functional groups also confer an overall surface charge when forming polyelectrolyte complexes. Acevedo-Fani et al. determined the ζ-potential of layer deposition in alginate–chitosan nanolaminates [76]. The overall surface charge changed depending on the charge of the polymer of the new layer, thus confirming its functional group’s presence. This is possible since at pH values 5.0–7.0, alginate solutions have a negative ζ-potential, increasing with polymer concentration. Meanwhile, the chitosan solutions are positively charged in the same pH range. Similar surface charge changes were observed by Picart et al. in polyelectrolyte multilayer poly(L-lysine)/hyaluronic acid films [77].

**Figure 9:**
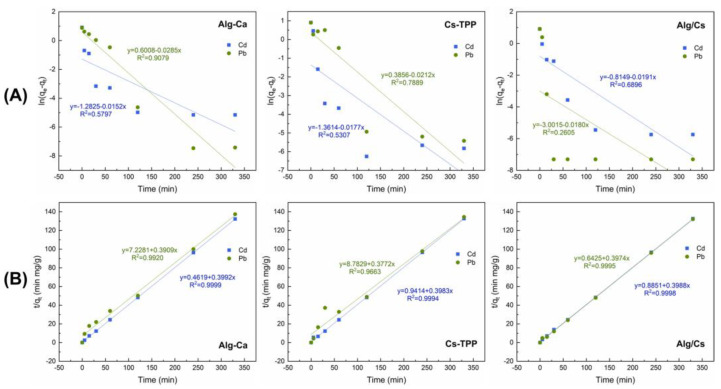
Fitting of experimental results for (**A**) pseudo-first- and (**B**) pseudo-second-order kinetic models.

Adsorption kinetics models were employed to investigate the rate and mechanism of the biosorption process. Figure 9 shows the fitting of the linear forms of the pseudo-first and pseudo-second-order kinetic models for the different films. Meanwhile, Table 1 shows the parameters determined for pseudo-first-order and pseudo-second-order models. The correlation coefficients (*R*^2^) indicate that Cd and Pb adsorption for all films follows the pseudo-second-order kinetic model. This model assumes that chemisorption is the primary mechanism involved in Cd and Pb biosorption; this includes electrostatic interactions, coordination bonding, and ion exchange [78]. Also, based on the adsorption rate constant (*k*_2_), the adsorption rate of Pb increases in Alg/Cs films and is higher than the Cd rate compared to Alg-Ca and Cs-TPP films.

Table 2 presents linearized Langmuir and Freundlich model parameters and correlation coefficient (*R*^2^) values. The Langmuir isotherm model considers monolayer adsorption with a homogeneous surface and homogeneous adsorption sites. On the other hand, the Freundlich model assumes multilayer adsorption with heterogeneous surfaces. According to *R*^2^ values, Cd and Pb adsorption onto biopolymeric films best fitted the Langmuir model, suggesting that the metals are homogeneously adsorbed onto the film surface. Regarding *q_max_*, the Pb adsorption capacity increases up to 159.74 mg/g for Alg-Cs hybrid films, compared to Alg-Ca and Cs-TPP samples, as seen before in removal efficiencies and kinetic modeling. This could be attributed to more available sites for adsorption from alginate and chitosan, such as amine, hydroxyl, and carboxyl. Interactions may be through electrostatic attraction, ionic exchange, or complexation [79]. Also, lead adsorption may be enhanced due to the three-dimensional network formed by intermolecular hydrogen bridges between alginate and chitosan, improving the hydrogel stability, accessibility, mechanical strength, and, therefore, ion adsorption [43,80].

### 2.6. Metal Recovery and Reuse of Films

Regeneration for reuse is essential for the cost-effectiveness of a biosorbent. Table 3 shows the results for the desorption of Cd and Pb retained on biosorbent gel films with 0.1 M HCl. It allowed very efficient Cd recovery from Alg-Ca and Alg/Cs hydrogel films after five consecutive adsorption/desorption cycles. The Cs-TPP film was only applied for one cycle before losing its integrity. Under acidic conditions, TPP reduces its ionization, which weakens its interaction with chitosan, resulting in a loss of hydrogel strength [81]. Jóźwiak et al. [82] reported damage in chitosan-TPP beads for reactive black five dye adsorption under acidic conditions. Nevertheless, Alg/Cs-based films could be reused through all cycles. Pb desorption efficiency was lower than Cd, even though adsorption efficiency remained >90% after five cycles. This may be attributed to the higher electronegativity of Pb compared to Cd, which leads to the most potent interaction of Pb with the biosorbent films. This tendency has been previously reported and attributed to the binding mechanisms since Pb interacts mainly through ion exchange. At the same time, Cd tends to form more labile interactions, mainly through electrostatic attraction. Pb can also form stable complexes, decreasing desorption efficiency; nevertheless, no complex formation was detected by FTIR spectra of films after adsorption (Figure S1) [83,84].

## 3. Conclusions

Biopolymeric alginate/chitosan-based gel films were synthesized by solving the casting method, and polyelectrolyte complex formation was characterized by its physicochemical properties. The combination of both polymers in Alg/Cs film decreased its swelling behavior compared to Alg-Ca film. Cd adsorption occurs more rapidly compared to Pb in all films. Biopolymeric Alg/Cs gel films showed higher adsorption of Pb ions in a shorter time (*t* = 15 min, 98%) compared to Alg-Ca and Cs-TPP films, mainly attributed to Pb’s hydrated ionic radius and hydration energy. The adsorption of both ions fitted a pseudo-second-order kinetic model and the Langmuir isotherm model, suggesting that the adsorption mechanism occurs through electrostatic interactions and ionic exchange processes. Desorption of Cd and Pb and reuse of Alg-Ca and Alg/Cs films were possible after five adsorption/desorption cycles. However, Pb exhibited lower desorption efficiency, consistent with its higher electronegativity. Based on this evidence, alginate/chitosan-based films are highly effective at removing Cd and Pb from water and may be reused, demonstrating their strong potential in potentially toxic metal removal. Further studies could be performed to study more effective Pb desorption and the competitive biosorption of these and other potentially harmful metals and emerging pollutants in water.

## 4. Materials and Methods

### 4.1. Film Preparation

Low-cost, high viscosity sodium alginate (SA) (Droguería Cosmopolita, S.A. de C.V., CDMX, MEX) and low-molecular-weight chitosan (Cs) (Sigma-Aldrich, St. Louis, MI, USA) were used. The preparation of the alginate–chitosan complex followed an approach like that reported by Bai et al. [63] to form composite gel spheres. The method was adapted to obtain gel films according to the following protocol: 1.0% (*w*/*v*) SA in deionized water and 1.5% (*w*/*v*) Cs in 2.0% (*v*/*v*) acetic acid solutions were prepared, 0.1 M calcium chloride (CaCl_2_) (Meyer, CDMX, MEX) and 10% (*w*/*v*) sodium tripolyphosphate (TPP) in 25% (*v*/*v*) ethanol (Meyer, CDMX, MEX) were used for SA and Cs crosslinking, respectively.

Films were obtained by the solvent casting method. SA and Cs solutions were poured on Petri dishes and dried at room temperature for 24 h to obtain SA and Cs films, respectively. SA dry film was immersed for 30 min in 0.1 M CaCl_2_, rinsed in deionized water to remove crosslinking excess, and dried at room temperature for calcium alginate film (Alg-Ca) obtention. Cs film was immersed for 5 min in 10% TPP solution for crosslinking, rinsed in deionized water, and dried at room temperature to obtain Cs-TPP film. For alginate/chitosan-based film (Alg/Cs), SA dry film was obtained as previously described and then immersed in a 1.5% Cs solution with 0.1 M CaCl_2_ for 30 min. The film was rinsed and dried under the same conditions.

### 4.2. Biopolymeric Films Characterization

#### 4.2.1. Fourier Transform Infrared Spectroscopy (FTIR)

The films’ FTIR spectra were obtained in a Nicolet iS50 spectrometer in attenuated total reflection mode (ATR) (Thermo Scientific, Waltham, MA, USA) with OMNIC™ 9.5 software (Thermo Scientific, Waltham, MA, USA). The spectrum was recorded in the 4000 to 500 cm^−1^ region using Origin Pro(version 2018) software (OriginLab Corporation, Northampton, MA, USA).

#### 4.2.2. Surface Analysis 

The surface of dry films was characterized by scanning electron microscopy (SEM) using a JEOL-JSM-5410LV microscope (JEOL USA Inc., Peabody, MA, USA) operating at 15 kV electron acceleration. Images of films were obtained before and after contact (*t* = 15 min) with 5.0 mg/L of cadmium and lead solution. Energy-dispersive X-Ray spectroscopy analysis confirmed cadmium and lead presence in films after the biosorption process.

Surface rugosity of biopolymeric Alg/Cs films before and after Cd and Pb adsorption (*t* = 15 min) was analyzed by atomic force microscopy (AFM) in a non-contact mode using Alpha 300RA equipment (WITec, QUL, DEU). Gel films were cut with a scalpel blade No. 11 and then attached to a glass microscope slide. The microscope probe had a spring constant of 42 N/m and a resonant frequency of 285 kHz. A 50 × 50 μm scan for each film was performed, and three 10 × 10 μm area scans were analyzed for rugosity value. The total scanning points per image were 65 536. The 2D and 3D images and their profiles were obtained with the WITec project Five 5.1 software (WITec, QUL, DEU).

#### 4.2.3. Thermogravimetric (TGA) and Differential Thermal Calorimetry (DSC) Analysis

A TGA/DSC analysis was performed to determine biopolymeric film materials’ thermal stability profile and decomposition temperature. Film samples were weighted in an ultra-micro scale (Autobalance AD6000, Perkin Elmer, Waltham, MA, USA) and placed in a simultaneous thermal analyzer STA6000 (Perkin Elmer, Waltham, MA, USA) using a nitrogen atmosphere, with a heating rate of 20 °C min^−1^. The TGA/DSC patterns were recorded from 30 to 600 °C.

#### 4.2.4. Swelling Studies

The swelling behavior of biopolymeric films was evaluated in deionized water at pH 6.5 at room temperature as swelling media. Each film sample was placed in a stainless-steel mesh and immersed in 50 mL of swelling media. At different time intervals, films were recovered. Excess water was blotted using delicate task wipers, and films were weighed. The swelling degree was calculated using the equation:

(1) $$Swelling\;\left(\%\right)=\frac{W_{s}-W_{d}}{W_{d}}$$

where *W_s_* is the weight of the swollen beads, and *W_d_* is the weight in the dried state.

Swelling changes in films at different pH levels (3, 4, 6.5, 8, 10) were also studied. To adjust the pH of the swelling media, 0.1 M NaOH and 0.1 M HCl were used. Dry films were weighed and immersed in each pH solution at room temperature. After five hours, the films were recovered and weighed. Swelling values were determined by Equation (1).

### 4.3. Batch Sorption Studies

#### 4.3.1. Cadmium and Lead Batch Removal

For batch sorption studies, working solutions at a concentration of 5 mg/L were obtained from a standard solution of 1000 mg/L (Sigma-Aldrich, St. Louis, MI, USA) for Cd and Pb, respectively. Then, 15 mL of each of the working solutions at pH 6.5 were individually exposed to 2 g/L of each film at different times (t = 0, 5, 15, 30, 60, 120, 240, 330 min) in Erlenmeyer flasks under orbital agitation (125 rpm) at room temperature. For isotherm analysis, adsorption was studied under the same conditions, varying initial ion concentration from 10 to 100 mg/L with a contact time of 6 h. Biosorbent films were recovered, and solution samples were acidified using 60 μL of HNO_3_ for the analysis of non-adsorbed metals by atomic absorption spectroscopy (AAS) (PinAAcle 900T, PerkinElmer Inc., Waltham, MA, USA). Cd and Pb removal efficiencies and adsorption capacities were calculated using the following equations:

(2) $$Removal\;efficiency\;\left(\%\right)=\left[\left(\frac{C_{i}-C_{t}}{C_{i}}\right)\right]\times 100$$

where *C_i_* is the metal ion’s initial concentration, and *C_t_* is the residual concentration at different contact times.

(3) $$Q_{t}=\left[\frac{C_{i}-C_{t}}{m}\right]\times V$$

where *C_i_* is the initial concentration of metal ions, *C_t_* is the concentration at different times, *m* is the biosorbent film’s mass, and *V* is the solution’s volume.

To analyze the Cd and Pb adsorption mechanism, pseudo-first-order (Equation (4)) and pseudo-second-order kinetics (Equation (5)) models were applied to experimental data using the linearized form of the equations.

(4) $$\ln\left(q_{e}-q_{t}\right)=\ln q_{e}-k_{1}t$$

(5) $$\frac{t}{q_{t}}=\frac{1}{k_{2}q_{e}^{2}}+\frac{t}{q_{e}}$$

where *q_e_* is the adsorption capacity at equilibrium, *q_t_* is the adsorption capacity at different times, *k*_1_ is the rate constant of the pseudo-first-order model, and *k*_2_ is the rate constant of the pseudo-second-order model.

For adsorption isotherms, the linearized forms of the Langmuir (Equation (6)) and Freundlich (Equation (7)) models were applied:

(6) $$\frac{1}{q_{e}}=\left(\frac{1}{{K_{L}\;q}_{max}}\right)\left(\frac{1}{C_{e}}\right)+\frac{1}{q_{max}}$$

(7) $$\log q_{e}=\log K_{F}+\left(\frac{1}{n}\right)\log C_{e}$$

where *K_L_* is the Langmuir constant, *q_e,max_* is the maximum adsorption capacity, *C_e_* is the equilibrium concentration of metal ion, and *K_F_* is the Freundlich constant.

#### 4.3.2. Desorption and Regeneration of Films

Desorption and reusing experiments were also conducted to investigate the reusability of the films as adsorbents. For this purpose, 2 g/L of films were applied to a 5.0 mg/L solution of each metal for 60 min, pH 6.5, 125 rpm. After the adsorption process, the film was placed in a flask containing 15 mL of 0.1 M HCl as a desorbing agent at 125 rpm using an orbital shaker for 15 min. The film was washed three times against water to remove any remaining acid and, later, suspended in a metal solution (5 mg/L) for the subsequent adsorption cycle. The adsorption-desorption cycle was carried out five times using the same film. AAS analyzed the non-absorbed metal concentration. The desorption efficiency of metal ions onto biopolymeric films for each cycle was calculated using Equation (8).

(8) $$Desorption\;efficiency\;\left(\%\right)=\frac{q_{des}}{q_{ads}}\times 100$$

where *q_des_* is the desorbed concentration, and *q_ads_* the adsorbed concentration of each metal on each cycle.

## Figures and Tables

**Table 1:** Kinetic parameters for cadmium and lead adsorption onto biopolymeric films.

Biosorbent Film	Pseudo-First-Order Model			Pseudo-Second-Order Model		
	*q_e_*	*k*_1_	*R* ^2^	*q_e_*	*k*_2_	*R* ^2^
**Alg-Ca**						
Cd	0.28	4.60 × 10^−5^	0.5797	2.51	0.3450	0.9999
Pb	1.82	−1.0 × 10^−4^	0.9079	2.56	0.0211	0.9920
**Cs-TPP**						
Cd	0.26	−5.37 × 10^−5^	0.5307	2.51	0.1685	0.9994
Pb	1.47	−1.0 × 10^−4^	0.7889		0.0162	0.9663
**Alg/Cs**						
Cd	0.44	−5.78 × 10^−5^	0.6896	2.51	0.1797	0.9998
Pb	0.05	−1.0 × 10^−4^	0.2605	2.52	0.2458	0.9995

**Table 2:** Isotherm parameters for cadmium and lead adsorption onto biopolymeric films.

Biosorbent Film	Langmuir			Freundlich		
	*R* ^2^	*q_max_*	*K_L_*	*R* ^2^	*1/n*	*K_F_*
**Alg-Ca**						
Cd	0.9875	198.02	0.0016	0.9702	0.9487	0.3704
Pb	0.9780	27.78	0.0080	0.8920	0.7338	0.4053
**Cs-TPP**						
Cd	0.9665	12.96	0.0302	0.8795	0.7907	0.6521
Pb	0.9858	19.26	0.0075	0.9438	0.7430	0.2592
**Alg/Cs**						
Cd	0.9944	60.98	0.0082	0.9515	0.8074	0.7175
Pb	0.9856	159.74	0.0029	0.9482	0.8174	0.6969

**Table 3:** Cadmium and lead desorption efficiencies with 0.1 M HCl for continuous film usage.

Cycle	Desorption Efficiency (%)					
	Alginate		Chitosan		Alginate–Chitosan	
	Cd	Pb	Cd	Pb	Cd	Pb
1	97.7	49.0	94.3	64.6	98.2	60.0
2	99.2	56.6	N/A *	N/A	99.9	65.9
3	99.5	79.7	N/A	N/A	99.8	71.8
4	99.9	82.3	N/A	N/A	99.9	75.5
5	99.9	79.4	N/A	N/A	98.8	77.6

* N/A: Not applicable.

## Data Availability

Data are contained within the article and Supplementary Materials.

## Supplementary Materials

The following supporting information can be downloaded at: https://www.mdpi.com/article/10.3390/gels10100669/s1, Figure S1: FTIR spectra of Alg/Cs based film, before and after Cd and Pb adsorption (t = 15 min, pH 6.5).
