# Supplementary material for: pH-Responsive Alginate/Chitosan Gel Films: An Alternative for Removing Cadmium and Lead from Water
Source: Gels. 2024 Oct 19;10(10):669. doi: 10.3390/gels10100669 (PMC11507177; doi:10.3390/gels10100669)
Supplement: Supplementary file 1 [file gels-10-00669-s001.zip › gels-3235250-supplementary.pdf]

pH-Responsive Alginate/Chitosan Gel Films: An Alternative for Removing Cadmium and Lead from Water

Silvia Carolina Moreno-Rivas [1], María José Ibarra-Gutiérrez [1], Daniel Fernández-Quiroz [1,*], Armando Lucero-Acuña [1], Alexel J. Burgara-Estrella [2] and Paul Zavala-Rivera [1,*]

[1] Department of Chemical Engineering and Metallurgy, University of Sonora, Hermosillo 83000, Mexico; carolina.moreno@unison.mx (S.C.M.-R.); majoibarragtz@gmail.com (M.J.I.-G.); armando.lucero@unison.mx (A.L.-A.)
[2] Department of Physics Research, University of Sonora, Hermosillo 83000, Mexico; alexel.burgara@unison.mx
* Correspondence: daniel.fernandez@unison.mx (D.F.-Q.); paul.zavala@unison.mx (P.Z.-R.)

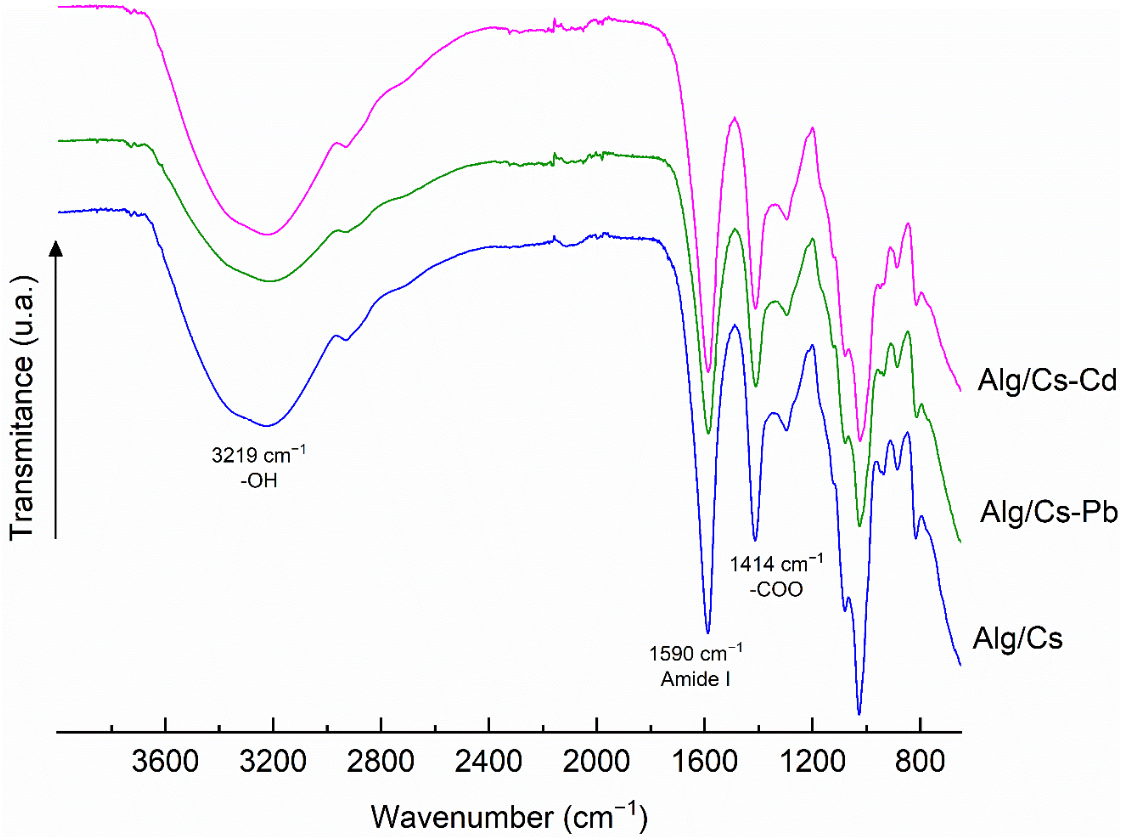

Figure S1. FTIR spectra of Alg/Cs based film, before and after Cd and Pb adsorption (t=15 min, pH 6.5).
